# Chemiluminescent organic nanophotosensitizer for a penetration depth independent photodynamic therapy†

**DOI:** 10.1039/d0ra01477j

**Published:** 2020-03-24

**Authors:** Xiaomei Lu, Xingwen Song, Qi Wang, Wenbo Hu, Wei Shi, Yufu Tang, Zizi Wu, Quli Fan, Wei Huang

**Affiliations:** Key Laboratory of Flexible Electronics (KLOFE), Institute of Advanced Materials (IAM), Nanjing Tech University (Nanjing Tech) Nanjing 211816 China iamwbhu@njtech.edu.cn iamwshi@njtech.edu.cn; Key Laboratory for Organic Electronics and Information Displays, Institute of Advanced Materials (IAM), Nanjing University of Posts & Telecommunications (NUPT) Nanjing 210023 China iamqlfan@njupt.edu.cn; Shaanxi Institute of Flexible Electronics (SIFE), Northwestern Polytechnical University (NPU) Xi'an 710072 China

## Abstract

Photodynamic therapy initiated by external photoexcitation is a clinically-approved therapeutic paradigm, but its practical application has been severely hindered by the shallow penetration of light. Here, we describe a penetration-independent PDT modality using a chemiluminescent organic nanophotosensitizer, which is activated by hydrogen peroxide instead of external photoexcitation.

## Abstract 2

A chemiluminescent organic nanophotosensitizer activated by hydrogen peroxide was fabricated for a potential penetration depth-independent photodynamic therapy.

## Graphical abstract

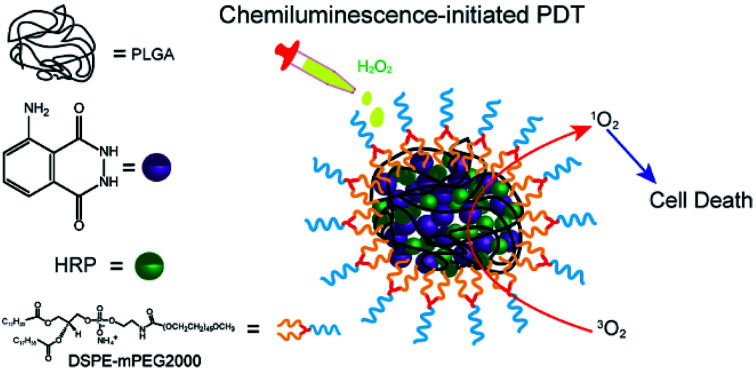

Photodynamic therapy (PDT) performed with the cooperation of a photosensitizer, molecular oxygen and light has become a minimally non-invasive therapeutic paradigm in clinics for the treatment of various diseases such as psoriasis, vitiligo and cancer.^1–4^ In general, exposing photosensitizers to suitable light, generates very toxic reactive oxygen species (mainly, single oxygen, ^1^O_2_) that kill tumour cells. Near-infrared light is preferred as external light to activate PDT owing to its considerably deeper penetration into tissue as compared to ultraviolet or visible light.^5,6^ However, advances in PDT have been severely confined to superficial lesions for decades as all these external light-based phototherapies, including PDT, suffer from rapid attenuation of external light in tissue.^7^ Recently, Cerenkov radiation has been used as external light to break the depth dependency of PDT.^7–9^ Although conceptually impressive, the expensive radiation source and inevitable DNA damage induced by ionizing radiation remain major limitations for practical applications. Therefore, it is highly desirable to develop novel better PDT with the penetration depth-independent feature.

Unlike external light sources, internal light sources, such as fibre-optic light sources, could address the penetration issue and have thus been proposed as an alternative solution to activate PS, but it brings invasive problems.^10^ In this case, another intriguing internal light arising from chemiluminescence has been proposed for penetration depth independent PDT. In this paradigm, chemiluminescence, which occurs when a specific chemical (such as luminol) is mixed with an appropriate oxidizing agent (such as hydrogen peroxide, H_2_O_2_), could activate the adjacent photosensitizer to proceed to PDT. Moreover, elevated H_2_O_2_ levels have been found in several types of cancer cells compared to that in normal cells, potentially affording H_2_O_2_-activatable PDT with good selectivity. However, only few chemiluminescent PDT systems have been reported.^11–14^ Moreover, these chemiluminescent PDT systems usually require coupling with additional PS to activate PDT by energy transfer, complicating the system with reduced reproducibility. In addition, to match the absorption of near-infrared absorptive PS (such as chlorin e6) for efficient energy transfer, quantum dots were usually employed to red-shift bioluminescence,^10,15^ which not only complicates the system but also raises the potential of metal-induced toxicity issues.

Herein, we fabricated a novel chemiluminescent NPs (C NPs) as a nanophotosensitizer for penetration depth independent PDT in tumour cells and bacteria (Scheme 1). This concise chemiluminescent NPs consisted of luminol and horseradish peroxidase (HRP), which is activated by hydrogen peroxide instead by external photoexcitation. In H_2_O_2_-rich conditions, C NPs exhibited remarkably enhanced ^1^O_2_ production compared to the luminol/HRP mixture. Finally, C NPs was demonstrated as a powerful nanophotosensitizer for efficient PDT in tumour cells and bacteria without external photoexcitation, becoming a promising platform for the future design of efficient PDT at high tissue depth.

**Scheme 1:**
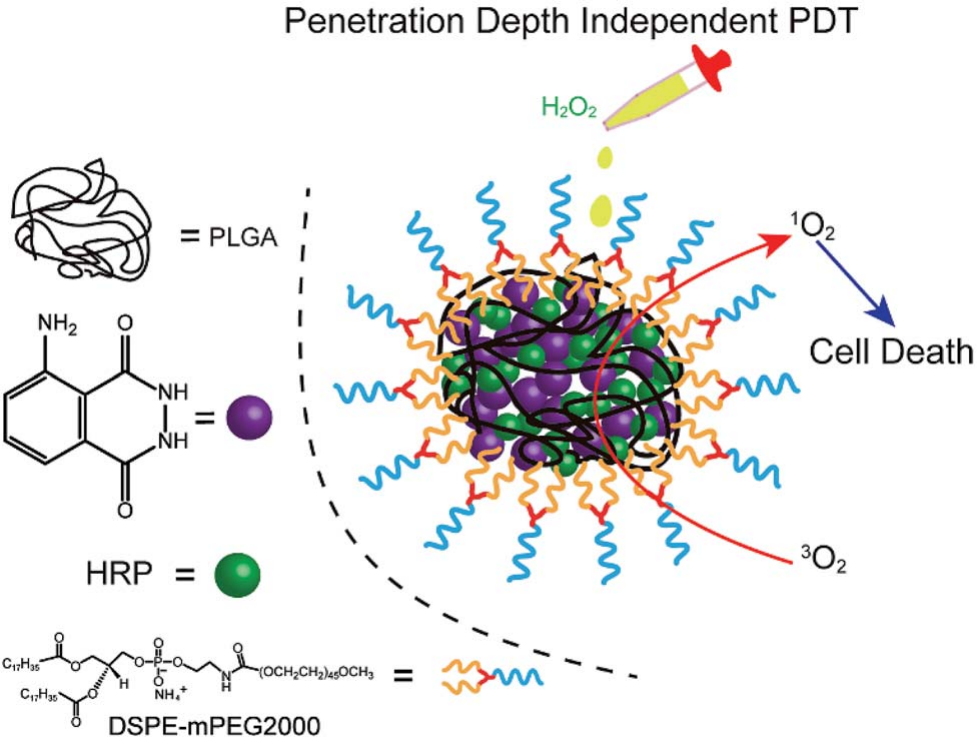
Schematic illustration of the NPs structure and penetration depth-independent PDT.

C NPs were prepared by the self-assembly of PLGA, luminol, HRP, and DSPE-mPEG2000 *via* a nanoprecipitation method (Scheme 1).^16^ The detailed preparation process is presented in the ESI.† Briefly, the PLGA was dissolved in acetonitrile. Luminol and HRP were dissolved in an aqueous solution, which was then added into the previous PLGA acetonitrile solution. This mixed solution was added dropwise to an aqueous solution of DSPE-PEG2000. After gently stirring for 4 h at room temperature, the remaining organic solvent and free molecules were removed by ultrafiltration. The designed C NPs could be stored in an aqueous solution up to 2 months without any eye-observed precipitation (Fig. 1a), indicating their excellent stability. Transmission electron microscopy (TEM) results reveal the spherical morphology of the C NPs with high monodispersity (Fig. 1b), while dynamic light scattering (DLS) indicated their average hydrodynamic diameter with a value of about 70 nm (Fig. 1c).

**Fig. 1:**
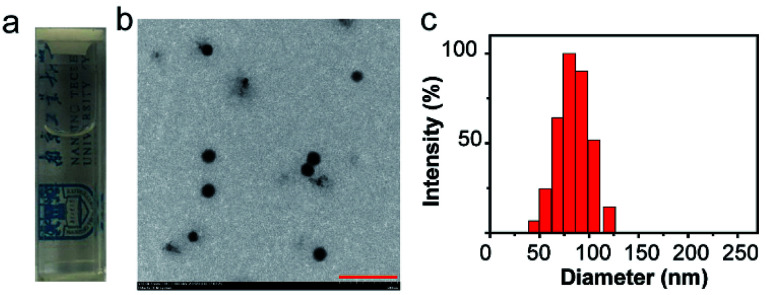
(a) Photograph of C NPs in an aqueous solution. (b) TEM image of C NPs. Scale bar: 500 nm. (c) Representative DLS of C NPs.

The production of reactive oxygen species (ROS, *e.g.*, ^1^O_2_) in NPs or luminol (L) + HRP was experimentally confirmed by the photodegradation of anthracene-9,10-diyl-bis-methylmalonate (ADMA) in the presence of H_2_O_2_ and molecular oxygen.^17,18^ After the addition of H_2_O_2_, the characteristic absorbances (260, 358, 378 and 399 nm) of ADMA dispersed in L + HRP solutions gradually decreased with prolonged time (Fig. 2a), indicating the inefficient production of ^1^O_2_. In sharp contrast, the absorbance of ADMA decreased remarkably in a mixture of ADMA and NPs after the addition of H_2_O_2_ (Fig. 2b). This is a solid evidence to illustrate that C NPs can be activated by H_2_O_2_ rather than external photoexcitation to perform PDT. Notably, within 2 min, the NPs almost totally consumed the AMDA, while the L + HRP only showed negligible consumption (Fig. 2c), which unambiguously demonstrated much more efficient production of ^1^O_2_ by C NPs. This is reasonable because L and HRP within NPs are close together, which is favourable for chemiluminescence. Despite the origin of ^1^O_2_ production during the C NP-induced chemiluminescence, the above-mentioned results clearly indicate that C NPs can efficiently produce ^1^O_2_ under H_2_O_2_ activation, which shows tremendous potential *in vivo* PDT in high tissue depth.

**Fig. 2:**
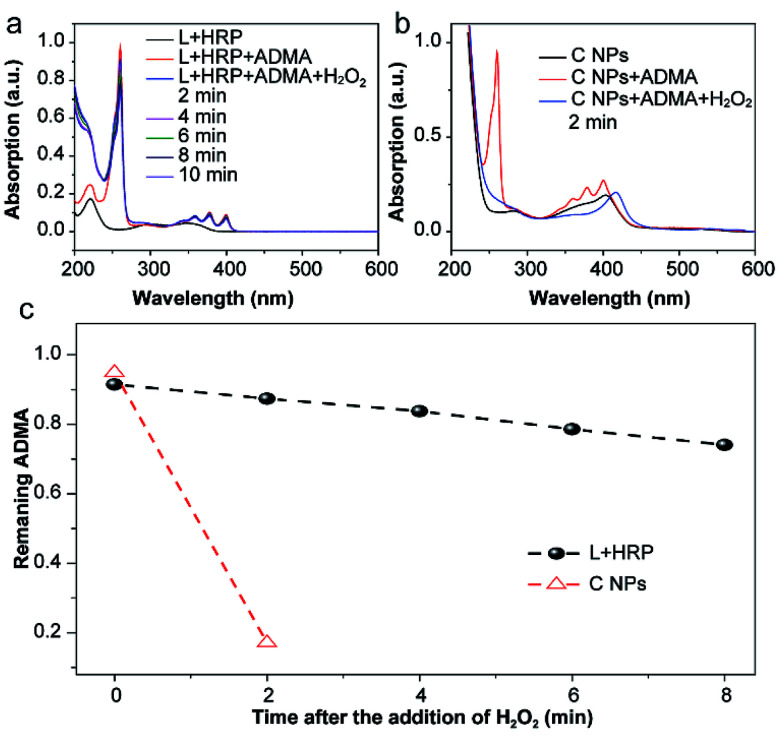
The absorption spectra of a C NPs (a) or L + HRP (b) and ADMA mixture before and after addition of H_2_O_2_. The spectra were obtained every 2 min after addition of H_2_O_2_. Rapidly decreased characteristic absorbance at 260 nm of ADMA within 2 min confirms the efficient ^1^O_2_ production of C NPs in the presence of H_2_O_2_. (c) Kinetic curves of the ADMA consumption of L + HRP and C NPs.

To verify the PDT effects, we utilized calcein-AM (living cell) and propidium iodide (PI, dead cell) cell viability kits to distinguish the dead cells from living ones (Fig. 3).^19,20^ After incubation with H_2_O_2_, H_2_O_2_ + HRP, and H_2_O_2_ + HRP alone, HeLa cells showed comparable cellular viability to the blank group (control) without any treatments, which demonstrate the resistance of HeLa cells toward H_2_O_2_, H_2_O_2_ + HRP, and H_2_O_2_ + HRP. Without the addition of H_2_O_2_, both L + HRP and C NP-incubated HeLa cells exhibited negligible cytotoxicity, suggesting low dark-cytotoxicity of L + HRP and C NPs. After the addition of H_2_O_2_, C NP-incubated HeLa cells exhibited strong cytotoxicity relative to the mixed solution of L + HRP.

**Fig. 3:**
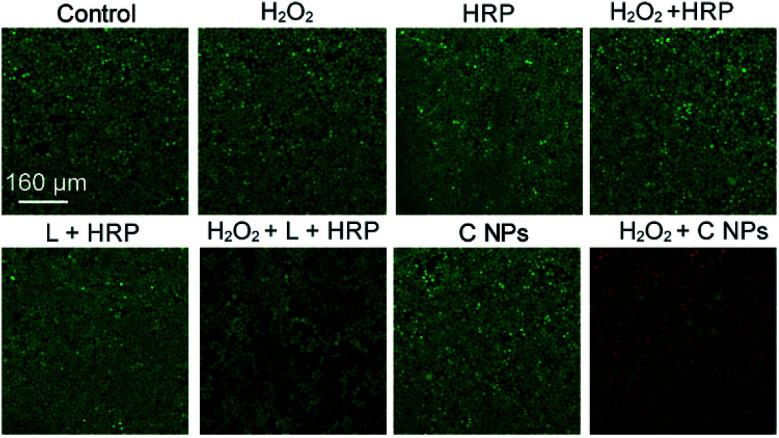
Live/dead assay of HeLa cells. Green colour represents live cells, and red colour represents dead cells.

Furthermore, we evaluated the antibacterial ability of NPs using Gram-negative bacteria, namely *E. coli*. As shown in Fig. 4a, all groups treated with C NPs, L + HRP, and H_2_O_2_ alone showed a tiny difference as compared to the blank group (control) without any treatment, which means that C NPs, L + HRP, and H_2_O_2_ have no obvious influence on *E. coli*. After the addition of H_2_O_2_, a huge decrease in C NPs + H_2_O_2_ was observed, while only a small decrease in L + HRP + H_2_O_2_, indicating a stronger antibacterial ability of C NPs. The antibacterial efficiency of NPs + H_2_O_2_ was determined to be 70% (Fig. 4b), which is approximately 10-fold stronger than L + HRP + H_2_O_2_ (7%). These results clearly demonstrated the strong antibacterial ability of C NPs.

**Fig. 4:**
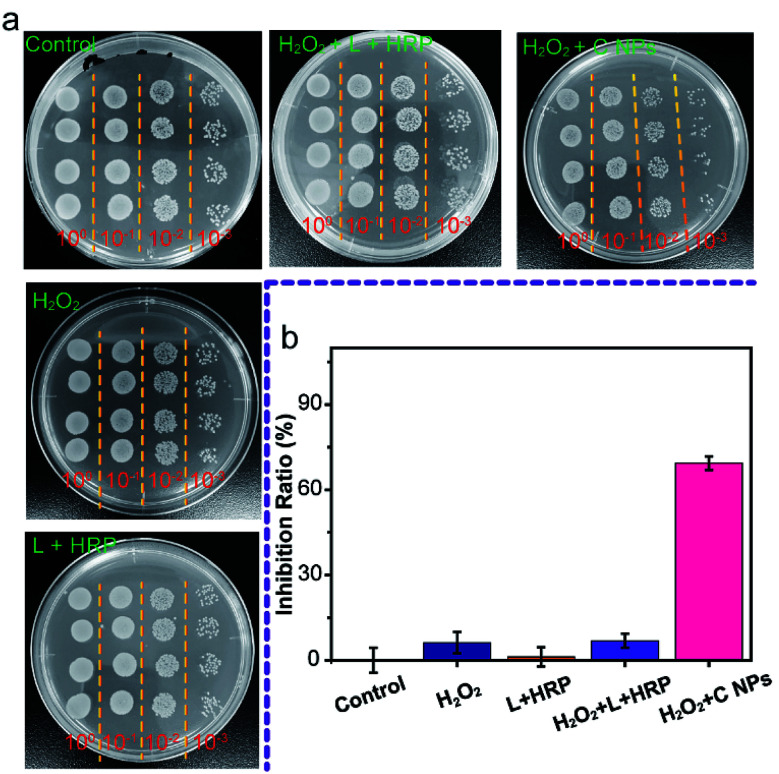
(a) Colony-forming units (CFU) for *E. coli* treated with NPs before and after addition of H_2_O_2_ on LB agar plate. The treated *E. coli* diluted 10^0^ to 10^−3^, 6 microliters of each dilution were inoculated to solid LB media, the *E. coli* were grown at 37 °C for 12 h. (b) Antibacterial activity of NPs before and after addition of H_2_O_2_.

In summary, we have fabricated a chemiluminescent organic nanophotosensitizer, namely C NPs, which could be activated by H_2_O_2_ instead by external photoexcitation. The C NPs show a very strong ^1^O_2_ generation ability in the presence of H_2_O_2_. The tumour cells and bacteria explanation clearly demonstrate that C NPs could be used as a powerful nanophotosensitizer for potential penetration depth-independent PDT. Our results provide an attractive platform for the future design of a powerful photosensitizer, which can expand the application scope of PDT.

## Conflicts of interest

There are no conflicts to declare.

## Supplementary Material

RA-010-D0RA01477J-s001
