# Supplementary material for: Chemiluminescent organic nanophotosensitizer for a penetration depth independent photodynamic therapy
Source: RSC Adv. 2020 Mar 24;10(20):11861–4. doi: 10.1039/d0ra01477j (PMC9050500; doi:10.1039/d0ra01477j)
Supplement: RA-010-D0RA01477J-s001 [file RA-010-D0RA01477J-s001.pdf]

Electronic Supplementary Material (ESI) for RSC Advances.
This journal is © The Royal Society of Chemistry 2020

Chemiluminescent organic nanophotosensitizer for penetration depth independent photodynamic therapy

Xiaomei Lu,[‡,a] Xingwen Song,[‡,a] Qi wang,[a] Wenbo Hu,*[a] Wei Shi,*[a] Yufu Tang,[a] Zizi Wu,[a] Quli Fan,*[b] and Wei Huang[a,b,c]

[a.] *Key Laboratory of Flexible Electronics (KLOFE), Institute of Advanced Materials (IAM), Nanjing Tech University (Nanjing Tech), Nanjing 211816, China. E-mail: iamwbhu@njtech.edu.cn; iamwshi@njtech.edu.cn*

[b.] *Key Laboratory for Organic Electronics and Information Displays, Institute of Advanced Materials (IAM), Nanjing University of Posts & Telecommunications (NUPT), Nanjing 210023, China. E-mail: iamqlfan@njupt.edu.cn*

[c.] *Shaanxi Institute of Flexible Electronics (SIFE), Northwestern Polytechnical University (NPU), Xi'an 710072, China*

‡ These authors contributed equally to this work.

Materials

All chemicals and reagents were purchased from Sigma-Aldrich or Fisher Scientific unless otherwise specified and were used without further purification. DSPE-mPEG2000 was obtained from Avanti (Alabaster, AL, USA). Live & Dead Viability Kit was purchased from KeyGEN BioTECH.

Instrument

Steady-state UV absorption spectrum data was obtained on a SHIMADZU UV-3600 PLUS UV-Vis-NIR spectrophotometer; Transmission electron microscopy (TEM) images were performed on a HT7700 transmission electron microscope operating at 100 kV. Dynamic light scattering (DLS) was performed on a particle size analyzer

(NanoBrook 90Plus, Brookhaven Instruments Corporation). Cell death images were observed with an inverted laser scanning confocal microscope (Zeiss LSM 710 Meta NLO).

Preparation of C NPs

C NPs were prepared by self-assembly of PLGA, Luminol and DSPE-mPEG2000 through a one-step nanoprecipitation method. Briefly, PLGA r was first dissolved in an acetonitrile solution (2 mg/ml). Then, the pH of 1 mg/mL Luminol aqueous solution was adjusted to 7 using NaOH solution, and 2 mg/mL HRP aqueous solution was added. Add the prepared PLGA acetonitrile solution to the Luminol/HRP aqueous solution to obtain a Luminol/ HRP/ PLGA solution. The solution of Luminol/HRP/PLGA was then added dropwise to the 2 mg/mL DSPE-mPEG2000 aqueous solution under gentle stirring. The mixed solution was sonicated for 3 minutes and then gently stirred at room temperature for 4 h. C NPs were obtained after high-speed centrifugal purification to remove residual organic solvents and free molecules.

Measurement of singlet oxygen generation

Luminol (2 mg/mL) and HRP (0.4 mg/mL) were added to an aqueous solution of a singlet oxygen indicator ADMA at 1 mg/mL, and the initial ultraviolet absorption was measured. Subsequently, H_2O_2 was added to the mixed solution, and the ultraviolet absorption spectrum was measured every 2 minutes. The operation method of the C NPs experimental group

is the same, that is, the added Luminol and HRP are replaced with C NPs. In this way, we can compare the efficiency of the Luminol-H_2O_2-HRP chemiluminescence system and C NPs to generate singlet oxygen by the decline rate of the UV absorption peak at ADMA 261 nm.

Live & dead cell assay

Hela cells were seeded in a confocal dish and incubated in DMEM medium containing 10% fetal bovine serum and 1% streptomycin/ penicillin at 37 °C, 5% CO_2 for 24 h. Then, the medium was aspirated, washed once with PBS, and treated with a medium containing 200 μM of Luminol-HRP-PLGA nanoparticles for 6 h, and incubate with 1μM calcein-AM (AM, labeled live cells) and propidium iodide (PI, labeled dead cells) probes for 15 min. Cell death images were then observed using an inverted laser scanning confocal microscope (Zeiss LSM 710 Meta NLO). At the excitation wavelength of 488 nm, the collection range of calcein-AM emission is 500-550 nm, and the collection range of PI emission is 600-650 nm.

Antibacterial effect

The *Escherichia coli* were used in analysis of antibacterial effect based on the morphological characteristics of the colonies. *Escherichia coli* were obtained from ATCC. *E. coli* were cultured in LB broth at 37° C until OD_{600}=0.6, and further add 0.1 mL of Luminol (2 mg / mL), 0.1 mL of HRP (0.4 mg / mL) or 50 μL of H_2O_2 (3%) to the 2ml bacterial

solution, respectively, furthermore continue to shake in the incubator for 4 h, and then diluted 10^{-0}, 10^{-1}, 10^{-2}, 10^{-3}, with sterile physiological saline. 6 microliters of each dilution were inoculated to solid Medium LB. The bacteria were grown at 37C for 12 h and their growth was monitored every 3h. At the same time, quantitative analysis of the bacteria after treatment by counting method. Take the average bacterial growth number (N_{Control}) of the control group as 100%, that is, the bacteriostatic rate (A) is 0%, and the average colony number (N_1) in the fourth column of the experimental group is obtained by counting. By formula:

$$A = \frac{N_{\text{Control}} - N_1}{N_{\text{Control}}} \times 100\%$$

The antibacterial effect of the experimental group was calculated. The antibacterial effect repeated four times.
